# Review of Management of Clinical Stage I Small Cell Lung Cancer: The Rising Role of Surgical Resection

**DOI:** 10.3390/cancers18111781

**Published:** 2026-05-29

**Authors:** Gabriella R. Rasmussen, Eric Klipsch, Kathryn E. Engelhardt

**Affiliations:** Division of Cardiothoracic Surgery, Department of Surgery, Medical University of South Carolina, Charleston, SC 29425, USA; rasmussg@musc.edu

**Keywords:** small cell lung cancer, stage I disease, surgical resection, lobectomy, mediastinal staging, limited-stage small cell lung cancer

## Abstract

**Background:** Small cell lung cancer (SCLC) is an aggressive malignancy that has traditionally been treated as a systemic disease, with surgery largely excluded from standard management. A small subset of patients, however, present with clinical Stage I disease (T1–2N0M0). With improvements in imaging, staging, and systemic therapy, local therapy warrants consideration. **Methods:** We performed a narrative review of the literature focused on clinical Stage I SCLC, prioritizing studies addressing epidemiology, tumor biology, diagnostic workup, staging, treatment approaches, and surveillance. Emphasis was placed on current guideline recommendations and contemporary retrospective data relevant to surgical and non-surgical local therapies. **Results:** Clinical Stage I SCLC is rare and is frequently upstaged with complete diagnostic evaluation, highlighting the need for thorough staging and pathologic confirmation of node-negative disease when surgery is considered. Even in presumed local disease, distant metastases are many times evident with a proper staging workup. Retrospective analyses suggest potential for long-term control of disease in carefully selected Stage I patients treated with surgical resection, particularly lobectomy, as part of multimodality therapy that includes adjuvant systemic therapy. For patients who are not surgical candidates, stereotactic body radiation therapy combined with systemic therapy is a reasonable alternative. The role of prophylactic cranial irradiation and optimal surveillance strategies in Stage I disease remain areas of uncertainty. **Conclusions:** Clinical Stage I SCLC affects a small and unique group of patients where traditional treatment strategies may need to be reconsidered. Taken together, retrospective evidence suggests a survival benefit for surgery in carefully selected patients, although prospective validation is lacking. Surgery warrants consideration in appropriately staged, operable patients, while recognizing the limitations of existing data and the need for further study in this rare population.

## Simple Summary

This review article serves as a recommendation for the management of clinical Stage I small cell lung cancer. In the past several decades, the role of surgery for the treatment of Stage I small cell lung cancer has been revisited, and operative resection now serves as an important component of multimodal therapy, demonstrating potential for long-term control of disease in this patient population. Additionally, advancements in diagnostic imaging and clinical staging techniques make clinical Stage I disease more readily diagnosed, further opening the door for operative intervention for these patients. Given that this represents a significant change from prior treatment strategies, it is important to create a framework to inform physicians that incorporates both the current literature and clinical experience from experts in the field to ensure that this unique cohort of patients receives optimal management.

## 1. Introduction

Small cell lung cancer (SCLC) has long been treated as a uniformly systemic disease, with surgery historically playing a small role in its management [1]. This paradigm is largely rooted in early trials comparing surgery with radiotherapy, which concluded that surgical treatment did not improve outcomes [2,3]. However, these studies were conducted in an era predating modern staging and systemic therapy, and since they were published, the treatment algorithm has expanded to include multimodal therapy, specifically the inclusion of surgical resection in the multidisciplinary management paradigm. At the time, patients were staged without positron emission tomography (PET), brain magnetic resonance imaging (MRI), or routine invasive mediastinal evaluation [4]. Consequently, many patients labeled as having “stage I” disease likely harbored unrecognized nodal or distant metastases, fundamentally confounding comparisons between treatment strategies [4,5,6]. Although the overall incidence of SCLC has declined in parallel with decreasing tobacco use, the disease remains aggressive and clinically significant [7]. Outcomes continue to be poor, and SCLC accounts for a disproportionate share of lung cancer mortality despite fewer diagnoses overall [1,7,8]. This highlights the importance of optimizing treatment strategies across all stages, including the relatively small subset of patients diagnosed with Stage I disease.

Advances in imaging, staging, and systemic therapy have since made it possible to reliably diagnose Stage I disease [5,6]. Modern diagnostic tools now allow for more accurate identification of true Stage I SCLC, while contemporary chemotherapy and immunotherapy address the systemic nature of the disease [9]. For example, the recently published ADRIATIC trial demonstrated the benefit of durvalumab as a component of systemic therapy in limited-stage SCLC [10]. Other evolving therapies include antiangiogenic agents, such as anlotinib, which have proven beneficial in non-small cell lung cancer (NSCLC) and may have a role in the treatment of SCLC, pending further investigation [11,12,13]. In this context, population-based data consistently suggest that selected patients with clinical Stage I SCLC who undergo surgical resection as part of multimodality therapy experience improved survival [14,15]. Despite these findings, surgery remains infrequently offered, reflecting persistent reliance on historical assumptions rather than contemporary data [16,17]. The current evidence supports an individualized treatment strategy based on patient fitness and tumor operability [15]. For operable patients, surgical resection followed by adjuvant chemotherapy and immunotherapy provides a potentially advantageous treatment pathway [18,19]. For patients who are not surgical candidates, definitive local therapy with stereotactic body radiation therapy (SBRT) combined with systemic therapy offers an alternative approach that preserves local control while addressing systemic risk [20]. Further supporting a stage-specific approach, emerging biologic data demonstrate that SCLC is not a single disease entity but rather comprises molecularly distinct subtypes with differing transcriptional programs and clinical behavior [21]. This heterogeneity challenges the long-held assumption that all SCLC behaves uniformly and raises the possibility that limited-stage disease may follow different trajectories across biologic subgroups [21,22]. Together, these insights in combination with recent advances in adjuvant treatment, namely the advent of immunotherapy, further justify reconsideration of management strategies in Stage I disease.

This review synthesizes contemporary evidence regarding staging, surgical and non-surgical treatment options, adjuvant therapy, and surveillance strategies, with particular emphasis on the role of surgery within a modern multimodality treatment framework.

## 2. Epidemiology

Of new lung cancer cases diagnosed in 2022, only 12% were SCLC, with approximately 6 cases per 100,000 standard population down from 8 cases per 100,000 in 2013, largely reflecting reduced tobacco use over time [7,23]. Population-based analyses report that a minority of patients with SCLC present with limited-stage disease with only 5% of new cases being specifically Stage I [1,16]. A recent analysis of the Surveillance, Epidemiology, and End Results (SEER) database found a decline in limited-stage SCLC diagnoses from 31.1% in 2000 to 26.4% in 2019, which is likely attributable to improved staging techniques with greater sensitivity for nodal disease and distant metastases [8]. Recent trends reveal that when combining all stages, the age-adjusted incidence-based mortality rate decreased from 6.6 per 100,000 in 2005 to 3.5 per 100,000 in 2025, but the 1-year survival showed little improvement, from 32.4% in 2000 to 34.5% in 2019 [24]. The 5-year survival for limited-stage disease also remains low, increasing from 10% in 1998 to only 15.6% in 2014 [8]. Overall, the long-term outcomes remain poor for all stages of SCLC, demonstrating the need for continued improvements in treatment options and management algorithms.

## 3. Pathophysiology

SCLC is a high-grade neuroendocrine malignancy defined by rapid growth and early dissemination [1,25]. Comprehensive sequencing studies demonstrate near-universal loss of the tumor suppressor genes TP53 and RB1, along with a high mutational burden and marked chromosomal instability [22]. Additional alterations affecting cell cycle regulation, DNA damage repair, and apoptotic pathways promote unchecked proliferation and resistance to cell death [22]. Together, these features help explain the rapid growth kinetics and early metastatic potential characteristic of SCLC [1,22]. Importantly, this capacity for dissemination appears to be intrinsic and present early in tumor development, rather than acquired over time [22]. As a result, micrometastatic disease may be present even in T1-2N0 tumors [1]. Additionally, although SCLC has historically been regarded as a biologically homogeneous disease, emerging data demonstrate meaningful molecular heterogeneity [21]. Distinct transcriptional subtypes, including ASCL1-, NEUROD1-, POU2F3-, and YAP1-driven tumors, have been described and are associated with differences in neuroendocrine differentiation and signaling pathways [21]. These findings challenge the traditional one-size-fits-all view of SCLC and suggest potential differences in therapeutic vulnerabilities. However, despite these molecular distinctions, all recognized subtypes retain aggressive behavior and a propensity for early spread [21,22]. Thus, biologic heterogeneity does not reduce the need for systemic therapy, even in limited-stage disease [1].

Clinical outcomes in Stage I SCLC further reinforce the need for both local and systemic treatment of disease. Patterns of recurrence following definitive local therapy, whether surgical resection or stereotactic body radiation therapy, are dominated by distant and central nervous system failures rather than isolated local relapse [14,20,26]. These observations show that local therapy alone is insufficient and systemic therapy plays a critical role in controlling occult disease [9,14]. The persistent vulnerability of the central nervous system, even in limited-stage SCLC, further informs post-treatment surveillance strategies and discussions regarding prophylactic cranial irradiation (PCI) versus imaging-based monitoring [19,26].

## 4. Presentation and Diagnosis

**Figure 1:**
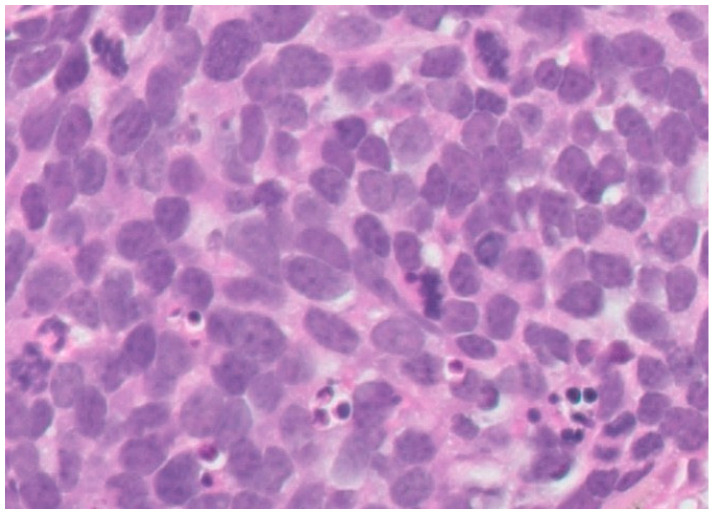
A representative light microscopy image of small cell lung cancer.

Patients with Stage I SCLC most commonly present with minimal or no symptoms, and Stage I disease is frequently identified incidentally [1,7]. Detection often occurs through lung cancer screening computed tomography (CT) scans or imaging performed for unrelated indications, and symptomatic presentations should raise suspicion of advanced or disseminated disease given the rapid growth and early dissemination characteristic of SCLC [1,7,22,27]. When symptoms are present, they are typically nonspecific and indistinguishable from other lung malignancies [1]. Patients may report cough, shortness of breath, chest discomfort, pleuritic pain, or hemoptysis. These features do not reliably differentiate SCLC from NSCLC [1]. Radiographically, Stage I SCLC most often appears as a solitary pulmonary nodule [16]. These lesions are frequently small (T1–T2) and typically lack obvious mediastinal lymphadenopathy on initial imaging [16]. SCLC cannot be distinguished from NSCLC based on imaging alone, and histological confirmation is required for a definitive diagnosis [1]. Tissue diagnosis is typically obtained via CT-guided percutaneous biopsy for peripheral lesions or endobronchial ultrasound (EBUS)-guided biopsy for more central tumors [1]. In select cases, surgical biopsy may be performed when suspicion for malignancy is high (e.g., PET-avid lesions in patients with significant smoking history) and less invasive approaches are nondiagnostic, which has been described in the literature [28]. The diagnosis of SCLC is based on characteristic morphologic features, classically described as small cells with scant cytoplasm, high mitotic activity, and nuclear molding, as seen in Figure 1, and is supported by immunohistochemical staining for neuroendocrine markers [29].

## 5. Staging of Disease

**Figure 2:**
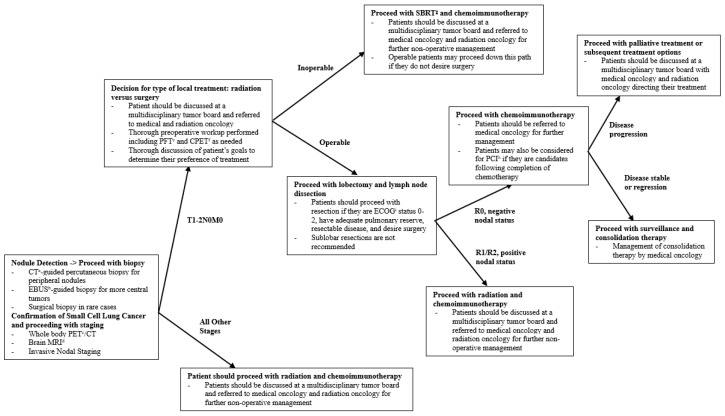
Treatment algorithm for the management of clinical stage 1 small cell lung cancer from the time of nodule detection through surveillance or disease progression. ^a^ Computed Tomography; ^b^ Endobronchial Ultrasound; ^c^ Positron Emission Tomography; ^d^ Magnetic Resonance Imaging; ^e^ Pulmonary Function Tests; ^f^ Cardiopulmonary Exercise Testing; ^g^ Stereotactic Body Radiation Therapy; ^h^ Prophylactic Cranial Irradiation; ^i^ Eastern Cooperative Oncology Group.

Following confirmation of SCLC, the next priority is a comprehensive staging workup prior to consideration of local therapy options. For reference, Figure 2 provides an outline of our recommended management of Stage I SCLC from the time of nodule detection to post-treatment follow-up. Historically, SCLC was staged using the Veterans Administration Lung Study Group (VALSG) system, which classified disease as either limited or extensive [30]. Limited-stage disease was defined as a tumor confined to one hemithorax and encompassed within a single tolerable radiation field, including ipsilateral mediastinal or supraclavicular lymph nodes [30]. Extensive-stage disease included tumor spread beyond a single radiation field, distant metastases, or malignant pleural or pericardial effusions [30]. While practical in the era of radiation-based treatment, this binary system lacked granularity and grouped biologically and prognostically distinct patients together [31]. Contemporary staging has shifted toward the tumor–node–metastasis (TNM) system, which has demonstrated prognostic relevance in SCLC and allows identification of a true limited-stage subset [5,6,31]. Stage I SCLC is defined as T1–2aN0M0 based on the 9th edition TNM classification of lung cancer, which differs from the 8th edition as outlined by the International Association for the Study of Lung Cancer, and according to the National Comprehensive Cancer Network (NCCN) guidelines, only patients with T1-2N0M0 disease are candidates for definitive local therapy such as surgery or SBRT as part of a multimodality approach [32,33,34].

As emphasized in the NCCN guidelines, SCLC is assumed to be a systemic disease until proven otherwise as Stage I presentations are frequently upstaged with higher T stages portending an increased risk of occult nodal metastases upon complete evaluation [32]. Given the propensity for early spread, we recommend expediting all components of the staging workup so as not to delay treatment. Recommended staging includes whole-body PET/CT to assess for mediastinal nodal involvement and distant metastatic disease, as well as brain MRI to exclude occult central nervous system metastases [4,32]. PET/CT plays a critical role in identifying otherwise unrecognized spread of disease and frequently results in stage migration that alters management [4]. In patients with suspected Stage I disease, imaging alone is insufficient to confirm node-negative disease and cannot reliably determine who is an appropriate candidate for local therapy. The NCCN recommends invasive mediastinal staging through either EBUS, endoscopic ultrasound (EUS), or mediastinoscopy to confirm true node-negative disease, recognizing the high risk of occult nodal involvement in SCLC [24]. EBUS is commonly the first-line invasive modality and allows sampling of mediastinal and hilar lymph node stations, including stations 2, 4, 7, 10, and 11 [35]. EUS fine-needle aspiration can complement EBUS by providing access to posterior and inferior mediastinal stations, namely stations 7, 8, and 9 [35]. Mediastinoscopy is reserved for cases in which EBUS- and EUS-guided biopsies are negative but clinical suspicion remains or sampling is inadequate [35]. When the diagnosis remains inconclusive and there are no signs of metastasis on imaging, surgical resection can provide definitive pathologic staging and help direct future treatment decisions [15]. Accurate staging is critical, as both surgical resection and SBRT are appropriate only in node-negative disease [32].

## 6. Treatment Strategies and Surveillance for Clinical Stage I Small Cell Lung Cancer

After the 1973 clinical trial published by Fox and Scadding, the standard treatment for SCLC shifted away from surgery and instead favored radiotherapy, and for many decades SCLC was not considered a surgical disease [2]. The current literature suggests that surgery can improve survival in Stage I SCLC, which can be more reliably diagnosed thanks to modern imaging and biopsy techniques [14,15,16,18,19,36,37,38,39,40,41,42,43]. Table 1 provides a list of contemporary studies that compare outcomes of surgery versus no surgery in SCLC. It is important to acknowledge that these studies are retrospective with limited sample sizes, which is partially a function of the overall low incidence of SCLC and even lower incidence of clinical Stage I SCLC. For this reason, their findings must be assessed with their inherent bias in mind, namely selection bias, confounding by indication, and unmeasured confounders. The conclusions of any individual study should be considered cautiously and put into the context of the greater literature pool as well as clinical experience. Additionally, in most of these studies, both the surgery and no surgery groups underwent multimodal therapy with some form of chemotherapy and/or radiation, again highlighting the importance of a multimodal approach in these patients. In accordance with these findings, current NCCN guidelines recommend surgery as an option in medically appropriate patients with Stage T1-2N0M0 biopsy-proven SCLC and negative pathologic lymph node staging [32]. The authors outline their recommendations for the management of clinical Stage I SCLC below.

As mentioned previously, many Stage I SCLC are diagnosed incidentally [1,7]. Upon identification of a suspicious lung nodule or mass, the next step would be a biopsy. Once SCLC is confirmed on biopsy, a full workup is performed, including PET/CT and brain MRI, and discussion at a multidisciplinary tumor board is mandatory. Additionally, if early stage is suspected, pulmonary function tests (PFT) should be performed at this time to help inform any potential operative plans. Provided there are no metastasis, pathologic lymph node staging is recommended next to rule out spread to the lymph nodes. Both EBUS and EUS are reasonable techniques for lymph node sampling, and we recommend sampling multiple mediastinal and hilar stations to reduce the chance of a false negative. If the sampling is inadequate or indeterminate, then mediastinoscopy should be performed for definitive pathologic analysis prior to consideration for local therapy. Any positive lymph nodes upstage the disease, precluding patients from proceeding with local therapy.

Patients who are clinically Stage I with negative pathologic nodal staging are candidates for local therapy, and the modality can be determined based on the patient’s preferences, performance status, and pulmonary reserve. Importantly, there is a gray area between operable and inoperable patients with no absolute cut off between them. Each patient should be discussed at a multidisciplinary tumor board, and only poor performance status (3–4) or inadequate pulmonary reserves should potentially preclude a patient from undergoing surgery. In patients with adequate reserve and good performance status (0–2), we recommend a surgical approach over SBRT if this is concordant with the patient’s goals. Data comparing the two treatment strategies is largely limited to retrospective studies of large national databases, which are subject to selection bias, but the current literature has shown improved survival for surgery as compared to radiation therapy alone [14,15,18,36,37,39,40,43]. Data has even shown improved outcomes in elderly patients when comparing surgery to radiotherapy [36]. Regarding the extent of resection, a lobectomy is recommended over a lobe-sparing approach given recent data demonstrating the superiority of lobectomy compared to sub-lobar resection, which has been demonstrated in multiple retrospective studies [16,18,19,37,38,44,45,46]. We do acknowledge that sub-lobar resections may be technically feasible in cases of peripheral lesions, which are rare in SCLC. However, compared to lobectomy, the chance of local recurrence is significantly higher for sub-lobar resections. For this reason, if patients cannot tolerate a lobectomy due to inadequate pulmonary reserve, confirmed on both PFT and cardiopulmonary exercise testing (CPET), then these patients should be considered for radiation therapy rather than resection for local control, sparing them the morbidity of an operation while providing equivalent long-term outcomes. For a lobectomy, patients must have FEV_1_ and DL_co_ values greater than 80% predicted. If these values are lower than 80% predicted, then a predicted postoperative value must be calculated based on the number of segments being resected. The predicted postoperative FEV_1_ and DL_co_ should be greater than 40%. If they do not meet the 40% threshold, CPET is performed, and any patients with a VO_2max_ below 10 mL/kg/min are too high-risk to undergo a lobectomy. Surgery should always include a thorough lymph node dissection or sampling. The current guidelines for lymph node sampling come from the American College of Surgeons (ACS) Commission on Cancer quality standard 5.8 and recommend sampling of at least one hilar and three mediastinal lymph node stations for adequate assessment of the lymph nodes [47]. Notably, lymph nodes sampled by EBUS or EUS are not included in the final pathology report according to quality standard 5.8 while those obtained during mediastinoscopy are [47]. In cases of SCLC, we believe the more lymph nodes sampled the better, given the propensity for early spread even in Stage I disease, and some studies have shown a survival benefit when more nodes are harvested [37]. Of note, the benefit of neoadjuvant chemoimmunotherapy is still debated and is not common practice in the United States. Its use may prove beneficial upon further investigation, and recent studies in Asia have assessed the use of chemoimmunotherapy in the neoadjuvant setting with promising results [48,49,50]. However, given that these strategies have not been formally tested within a Western cohort, in cases of borderline resectable disease, we advocate for proceeding with definitive chemoradiation with immunotherapy. Nonetheless, we also encourage patients to participate in ongoing clinical trials as appropriate to help determine how neoadjuvant treatment may contribute to the treatment algorithm.

Following surgery, patients with an R0 resection and N0 nodal status on final pathology do not require local radiotherapy, and they should proceed with systemic therapy after they have recovered from their operation [32,51,52]. Typically, we advocate waiting four to six weeks prior to initiating adjuvant treatment, but this timeframe can vary greatly based on each patient’s postoperative course. Additionally, it is worth emphasizing that all of these patients require multidisciplinary care, and all adjuvant treatment should be administered by medical and radiation oncologists. In R1 or R2 resections or in patients with R0 resections but nodal involvement, radiation therapy should be administered concurrently with systemic therapy [32,53,54,55,56]. Of note, the same radiotherapy schedule applies to those who have clinical Stage I disease but are not surgical candidates. As seen in the CONVERT trial and according to the American Society for Radiation Oncology, the preferred treatment is twice-daily rather than daily administration of radiation, starting with cycle 1 or 2 of chemotherapy and typically lasting three weeks [55,57]. The start time and duration of radiotherapy can vary from patient to patient under the direction of their radiation oncologist based on their ability to tolerate the treatment or other extenuating circumstances [55]. Additionally, for patients who are clinically N0 but pathologically N2, mediastinal radiation is conditionally recommended [55]. All patients should receive systemic chemotherapy and immunotherapy following resection under the guidance of a medical oncologist. The first-line treatment is the same for patients with Stage I-III disease, so even those with R1 or R2 resections or positive nodal disease on final pathology should receive similar chemotherapy regimens. The standard of care is four to six cycles of cisplatin and etoposide with a planned cycle length of every 21–28 days during concurrent radiation therapy [32,57]. Alternative agents and administration schedules exist, and the preferred treatment may vary based on institution or provider specific practices [32]. Additionally, current guidelines support the use of durvalumab as consolidation therapy [10,32]. Notably, the ADRIATIC trial, which demonstrated the benefit of durvalumab, only included patients with Stage I–III medically inoperable disease, and when published, the maturity of its data for progression-free survival was approximately 60%. The survival benefit was primarily seen in patients with Stage III SCLC, and, given no operable patients were included, the role of durvalumab following surgical resection remains debated [10]. Additionally, following completion of local therapy and systemic chemotherapy, patients with a good response or those with stable disease may potentially continue durvalumab until disease progression or intolerable toxicity for a maximum of 24 months [10,32]. In the unfortunate case that the disease progresses despite initial treatment, subsequent systemic therapy versus palliative therapy options should be discussed with the patient as directed by medical and radiation oncology.

For a select group of patients with complete or partial response following their initial treatment, PCI may provide some survival benefit since SCLC commonly metastasizes to the brain. The use of PCI became common after a meta-analysis published in 1999 in the *New England Journal of Medicine* showed that it improved overall and disease-free survival for SCLC patients in complete remission, and its benefit was again confirmed in a randomized control trial published in 2002 [58,59]. However, the role of PCI for Stage I patients in remission following surgical resection is still not completely understood [60,61]. The American Society for Radiation Oncology conditionally does not recommend PCI for Stage I SCLC, but they also recognize the available data is limited and suggest shared decision making with patients [55]. Table 2 includes the current literature, all of which comprises retrospective studies, detailing outcomes of PCI following surgery for Stage I SCLC [43,62,63,64,65]. Since most studies group all patients with resectable disease together, it is difficult to determine the benefit for Stage I specifically. Overall, the results of these studies are mixed with some finding no statistically significant difference while others demonstrate a survival benefit. Notably, PCI should be performed after a restaging brain MRI is obtained and prior to initiation of durvalumab consolidation therapy [32,55]. Importantly, older patients and those with poor performance status are at an increased risk for neurotoxicity from PCI [32,66,67]. With this in mind, we recommend considering PCI in young patients who have R1 or R2 resections or nodal involvement following resection with good performance status (0–2) after a thorough risk–benefit discussion. However, all decisions for PCI should be made under the guidance of a radiation oncologist after multidisciplinary discussion of the risks and benefits of treatment.

Following the initial treatment, we recommend standard surveillance in accordance with the NCCN guidelines, which can vary to some degree based on individual institutional practices [32]. In general, patients should be seen every three months for the first two years, every six months the third year, and annually after that point. Blood work is only required as clinically indicated. CT chest, abdomen, and pelvis with contrast as well as a brain MRI regardless of PCI status should be performed at three-month intervals for the first year following treatment and at six-month intervals for the second year. After the second year, CT imaging can be performed every six to twelve months, and MRI is performed only as clinically indicated. A brain MRI is preferred over a CT head with contrast unless contraindicated. Additionally, routine PET scans are not necessary unless the contrasted chest, abdomen, and pelvis CT is contraindicated.

## 7. Conclusions

SCLC outcomes remain poor but are slowly improving, and surgery now plays an important role in medically operable patients with Stage I disease [32]. Given its aggressive nature and propensity for early spread, a thorough staging workup is essential prior to any treatment decisions, and all patients should be discussed at a multidisciplinary tumor board. For those patients who are unable to undergo resection or who have positive margins or pathologically positive nodes, radiation therapy is a key component of their treatment. Additionally, medical oncologists play an essential role in management since all patients require systemic therapy. PCI remains a topic of debate, but it may improve outcomes in select patients. There are still several gaps in the literature that warrant further investigation to optimize the treatment strategy for Stage I SCLC. Notably, the exact role of radiation therapy and PCI in patients who have undergone surgical resection is still uncertain as well as the potential role of neoadjuvant chemoimmunotherapy. Furthermore, select patients who undergo surgery may be candidates for de-escalation strategies. To further delineate the role of surgery within the treatment algorithm, prospective cohort studies or randomized control trials will be required. With the overall low incidence of SCLC compared to other lung cancers, inter-institutional collaboration will be key to accruing the necessary number of patients needed to definitively answer some of the pending questions related to Stage I SCLC treatment. Additionally, treatment strategies will continue to evolve as new chemotherapy and immunotherapy regimens become available. Notably, antiangiogenic agents and biomarker-guided treatment selection have shown promising results in NSCLC patients and may prove beneficial for SCLC as well [11,12,13,68]. Providers should regularly reevaluate their own practice to ensure that they are up to date with the most recent recommendations.

## Figures and Tables

**Table 1:** Outcomes of surgery (S) versus no surgery (NS) for small cell lung cancer.

Publication (First Author, Year)	Data Source (Timeframe)	Patient Population	Median Survival (Months)	5-Year Overall Survival (%)
Liu, 2023 [36]	SEER ^a^ (2004–2018)	Stage T1-T2N0M0, ≥70 years	S ^b^: 32 NS ^c^: 20	S: 30.6 NS: 17.6
Uprety, 2020 [43]	NCDB ^d^ (2004–2013)	Stage I	S with PCI ^e^: 93.0 S without PCI: 61.7 NS: 31.2	Not reported
Ahmed, 2017 [37]	SEER (2007–2013)	Stage I	S: 50 S ^†^: >60 NS: 16 NS ^‡^: 27	Not reported
Wakeam, 2017 [14]	NCDB (2004–2013)	Stage I-IIIA	S: 32.4 NS: 20.2	Not reported
		Stage I	S: 38.6 NS: 22.9	Not reported
Yang, 2018 [15]	NCDB (2003–2011)	Stage cT1-T2N0M0	S: 54.9 NS: 25.9	S: 48.1 NS: 28.3
Combs, 2015 [38]	NCDB (1998–2011)	Stage I	Not reported	S: 38 S *: 51 NS: 18
		Stage II	Not reported	S: 5 S *: 25 NS: 16
		Stage IIIA	Not reported	S: 20 S *: 18 NS: 12
Zhang, 2014 [39]	Single Center (1995–2013)	Stage I–IIIA	S: 30.5 NS: 16.9	Not reported
		Stage I	S: 25.8 NS: 22.5	Not reported
Varlotto, 2011 [18]	SEER (1988–2005)	Stage I	Lobectomy: 50 Sub-lobar: 30 NS: 20	Lobectomy: 47.4 Sub-lobar: 28.5 NS: 17.2
Schreiber, 2010 [16]	SEER (1988–2002)	Stage T1-T2Nx-N0M0	S: 42 NS: 15	S: 44.8 NS: 13.7
		Stage T3-T4N0M0 or Stage T1-T4N1-N2M0	S: 22 NS: 12	S: 26.3 NS: 9.3
		Stage T1-T4Nx-N2M0	S: 28 NS: 13	S: 34.6 NS: 9.9
Rostad, 2004 [41]	Cancer Registry of Norway (1993–1999)	Stage IA–IB	Not reported	S: 44.9 NS: 11.3
Badzio, 2004 [42]	Single Center (1984–1996)	Stage I–IIIA	S: 22.3 NS: 11.2	Not reported
		Stage I	S: 28 NS: 13	Not reported
		Stage II	S: 17 NS: 12	Not reported
		Stage III	S: 17 NS: 8	Not reported

^a^ SEER, Surveillance, Epidemiology, and End Results database; ^b^ S, Surgery; ^c^ NS, No Surgery; ^d^ NCDB, National Cancer Database; ^e^ PCI, Prophylactic Cranial Irradiation. ^†^ The S patients in this study who received radiotherapy were separated from those who did not. ^‡^ The NS patients in this study who received radiotherapy were separated from those who did not. * The S patients in this study who received additional treatment modalities were separated from those who did not.

**Table 2:** Outcomes of Prophylactic Cranial Irradiation (PCI) versus No Prophylactic Cranial Irradiation (NPCI) for Stage I Small Cell Lung Cancer Patients Following Surgical Resection.

Publication (First Author, Year)	Data Source (Timeframe)	Cohort Size	Cerebral Recurrence (%)	Median Survival (Months)	PCI Hazard Ratio (95% Confidence Interval)
Uprety, 2020 [43]	NCDB ^a^ (2004–2014)	PCI ^b^: 188 NPCI ^c^: 561	Not reported	PCI: 93.0 NPCI: 61.7	0.75 (0.58–0.98)
Lou, 2020 [62]	Single Center (2006–2017)	PCI: 46 NPCI: 100	PCI: 10.9 NPCI: 10.0	Not reported	0.95 (0.52–1.75)
Chen, 2018 ^†^ [63]	Single Center (2003–2015)	PCI: 5 NPCI: 12	PCI: 0 NPCI: 16.6	PCI: 38.0 ^‡^ NPCI: 29.8 ^‡^	0.33 (0.114–0.954) ^‡^
Xu, 2017 [64]	Single Center (2006–2014)	PCI: 19 NPCI: 59	PCI: 13.6 NPCI: 10.5	Not reported	1.61 (0.68–3.83)
Zhu, 2014 ^†^ [65]	Single Center (2003–2009)	PCI: 17 NPCI: 32	PCI: 9.0 ^‡^ NPCI: 22.2 ^‡^	PCI: 58.3 NPCI: 74.4	Not reported

^a^ NCDB, National Cancer Database; ^b^ PCI, Prophylactic Cranial Irradiation; ^c^ NPCI, No Prophylactic Cranial Irradiation; ^†^ Survival in these studies were reported as 5-year overall survival (%) ^‡^ These results included patients with Stage II–III disease.

## Data Availability

No new data were created or analyzed in this study. Data sharing is not applicable to this article.
